# A Novel Sensor Prototype with Enhanced and Adaptive Sensitivity Based on Negative Stiffness Mechanism

**DOI:** 10.3390/s20164644

**Published:** 2020-08-18

**Authors:** Lijun Liu, Yongzhong Nie, Ying Lei

**Affiliations:** 1 Department of Civil Engineering, Xiamen University, Xiamen 361005, China; liulj214@xmu.edu.cn; 2 FATRI (Xiamen) Technologies Co., Ltd., Xiamen 361000, China; bill.nie@fatritech.com

**Keywords:** sensor, negative stiffness, weak signal, adaptive sensitivity, micro controller, feedback control, adaptive amplification

## Abstract

Loess–mudstone/soil-rock interfacial landslide is one of the prominent landslide hazards that occurs in soil rock contacting zones. It is necessary to develop sensors with high sensitivity to weak and low frequency vibrations for the early warning of such interfacial landslides. In this paper, a novel monitoring sensor prototype with enhanced and adaptive sensitivity is developed for this purpose. The novelty of the sensitive sensor is based on the variable capacitances and negative stiffness mechanism due to the electric filed forces on the vibrating plate. Owing to the feedback control of adjustable electrostatic field by an embedded micro controller, the sensor has adaptive amplification characteristics with high sensitivity to weak and low frequency input and low sensitivity to high input. The design and manufacture of the proposed sensor prototype by Micro-Electro-Mechanical Systems (MEMS) with proper packaging are introduced. Post-signal processing is also presented. Some preliminary testing of the prototype and experimental monitoring of sand interfacial slide which mimics soil–rock interfacial landslide were performed to demonstrate the performance of the developed sensor prototype with adaptive amplification and enhanced sensitivity.

## 1. Introduction

Loess–mudstone/soil-rock interfacial landslide is one of the most prominent landslide hazards that occurs in soil rock contacting zones in the world [1]. In the loess–mudstone zones, the mudstone strata are covered by loose loess sediments. The contact characteristics of the mantle deposit between the loess-sediments and underlying mudstone strata provide a precondition in the formation and the development of interfacial landslides [1,2,3,4]. Many researchers have carried out numerical simulation, prediction models, and experimental study on landslides, and sliding mechanism [5,6,7]. Though research into landslide disasters is relatively extensive and in-depth, the false alarm rate is still high [8]. With the increasing number of seismic sensors deployed worldwide and the development of sensor technology, it has become possible to use seismic sensors for landslide monitoring, especially at a regional scale [9]. Much research have been done on landslide monitoring by all kinds of high precision sensors [10,11,12,13]. Recent studies show that large landslides can generate strong low-frequency seismic signals [14]. Many actual landslides have been detected with low-frequency seismic signals [12,15,16,17,18,19]. For instance, the main frequency of the weak seismic signal of the Xiaolin landslides occurring in Taiwan in 2009 was 0.5–1.5 Hz [17,18]. The predominant frequency band of landslides induced by typhoon Morakot in Taiwan in 2011 was 0.5–5 Hz [19]. The onset of the large amplitude low-frequency motion is earlier than the initiation of the observable high-frequency motions, and the low-frequency component shows the peak amplitude significantly earlier than the peak amplitudes of the high-frequency component. Thus, latent forthcoming landslides can be forecasted through infrasound monitoring by advancements in ultrasensitive low frequency sensors.

For monitoring weak and low frequency vibration signals, various types of advanced sensors have been developed. According to the realization principles, the sensors can be divided into: magnetoelectric sensors [20], piezoelectric sensors [21,22,23,24,25], fiber sensors [26,27], capacitive sensors, piezoresistive sensors etc. [28,29]. Sensors based on magnetoelectric technology are simple in design, but the larger size and great bulk are not conducive to a large number of installations. Piezoelectric type sensors have the characteristics of the good reliability, small size, light weight and wide frequency range, but find it difficult to achieve ultra-low frequency vibration measurement. Fiber sensors have been developed rapidly, with the characteristics of strong anti-electromagnetic interference, high sensitivity, light weight, small size and soft features, but the fiber is easily damaged. Variable capacitance and piezoresistive sensors can achieve small size and low frequency characteristics based on MEMS technology, but it is difficult to achieve ultra-low frequency because the mass cannot be too large. The sensitivity sensors for monitoring seismic wave are too bulky, consume large amounts of energy, and are expensive; for example, a Streckeisen STS-2 seismometer weighs 9 kg and costs more than USD 10,000, which can hardly meet the demands for a large number of sensitive sensors for monitoring large size landslides. Most of current sensors do not have adaptive sensitivity, i.e., sensors with high sensitivity to weak signals, intended for ambient monitoring, will saturate during strong excitation.

Some researchers have attempted to develop sensors with negative stiffness to detect the low frequency and weak vibration. Song et al. [30] proposed a weak force signal detection scheme based on negative stiffness and demonstrated that the proposed sensor shows higher sensitivity for weaker stimulus. Zheng et al. [31] studied a capacitive accelerometer with positive and negative feedback systems based on negative stiffness and adaptation, which possesses high sensitivity to low frequency and small input and a broad dynamic range. Lee and Park [32] studied the mechanical model that demonstrates a shift in the high-sensitivity region due to the interplay of negative stiffness and an adaptation mechanism. The authors have also studied the mechanism of a hair cell bioinspired sensor with ultra sensitivity to weak and low frequency vibration signals based on the gating model with negative stiffness of gating spring [33] and a bio-inspired tilt sensor model with adaptive gain and enhanced sensitivity the due to the negative stiffness mechanism implemented by magnet forces [34]. These studies have established theoretical foundations for the design of enhanced sensors with adaptive amplification and high-sensitivity, especially in the design of such sensors with small sizes by Micro-Electro-Mechanical System (MEMS).

In this paper, based on the previous theoretical studies, a novel sensor prototype with high sensitivity to weak and low frequency vibrations was developed for monitoring interfacial landslides. The adaptive and enhanced sensitivity is due to the negative stiffness effect which is implemented by a micro controller with an adjustable electrostatic field for measuring resultant force on the vibrating plate through feedback control. The manufacture of the sensor by MEMS including packaging was introduced. Some preliminary testing and experimental monitoring of sand interfacial slide were used to demonstrate the developed sensor prototype with adaptive amplification and enhanced sensitivity.

The rest of the paper is organized as follows: Section 2 investigates the mechanisms of the sensitive sensor. Section 3 describes the design, manufacture and packaging of the sensor prototype. Section 4 presents some preliminary testing of the sensor prototype. Section 5 explores the experimental monitoring of sand interfacial slide by the sensor prototype. Finally, Section 6 summarizes some conclusions of the research work.

## 2. The Mechanism of the Sensitive Sensor Prototype Based on Negative Stiffness

### 2.1. The Traditional Linear Model with Positive Stiffness Mechanism

Many kinds of traditional sensors are based on linear vibration with positive stiffness, which usually can be described as “mass-spring-damping” mechanical resonant systems. The positive stiffness mechanism is that the greater the deformation of the system, the greater the resistance. Under the action of base acceleration, the motion equation of the mass in an acceleration sensor is expressed as:

(1) $$m\ddot{x}+c\dot{x}+kx=-m{\ddot{x}}_{g}\left(t\right)$$

where *m*, *c* and *k* are the mass, damping and stiffness of the system, respectively, $\ddot{x}$, $\dot{x}$ and x are the relative acceleration, velocity and displacement of the mass, respectively, and ${\ddot{x}}_{g}$ is the ground acceleration.

Then, the amplification factor is expressed as:

(2) $$\mu =\frac{A_{m}}{A_{s}}=\frac{1}{\sqrt{{\left[1-{\left(\frac{\omega }{\omega_{n}}\right)}^{2}\right]}^{2}+{\left[2\zeta \left(\frac{\omega }{\omega_{n}}\right)\right]}^{2}}}$$

where $A_{m}$ and $A_{s}$ are the dynamic and the static output, respectively, $\omega_{n}$ is the natural frequency of the system, ω is the frequency of the ground acceleration, and ζ is the damping ratio. At low frequency, the acceleration component is very weak and the output of the sensor is very faint. If the mass block is not increased, the low-frequency signal could be submerged by the measurement noises.

**Figure 1:**
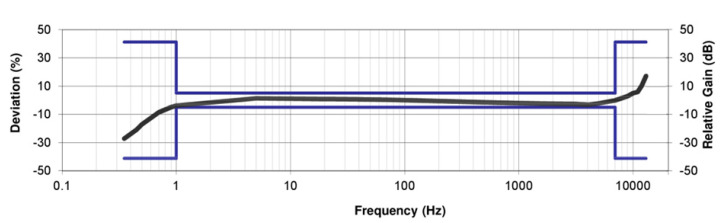
The typical amplitude response curve of a traditional acceleration sensor with high sensitivity (provided by FATRI (Xiamen) Technologies Co., Ltd.).

Figure 1 is the typical amplitude response of an acceleration sensor with high sensitivity produced by FATRI (Xiamen) Technologies Co., Ltd. The calibration sensitivity is 10 V/g when the test level is 0.1 g. Obviously, in this frequency sweep, the sensitivity begins to decrease after less than 5 Hz. Due to the poor low-frequency response of the system or the poor low-frequency signal-to-noise ratio of the system, under low-frequency excitation, the acceleration component is weak, and the low-frequency signal will be submerged by the measurement noise. So, the sensitivity of the sensor at low frequencies will decrease.

### 2.2. The Proposed Model with Negative Stiffness Mechanisms

**Figure 2:**
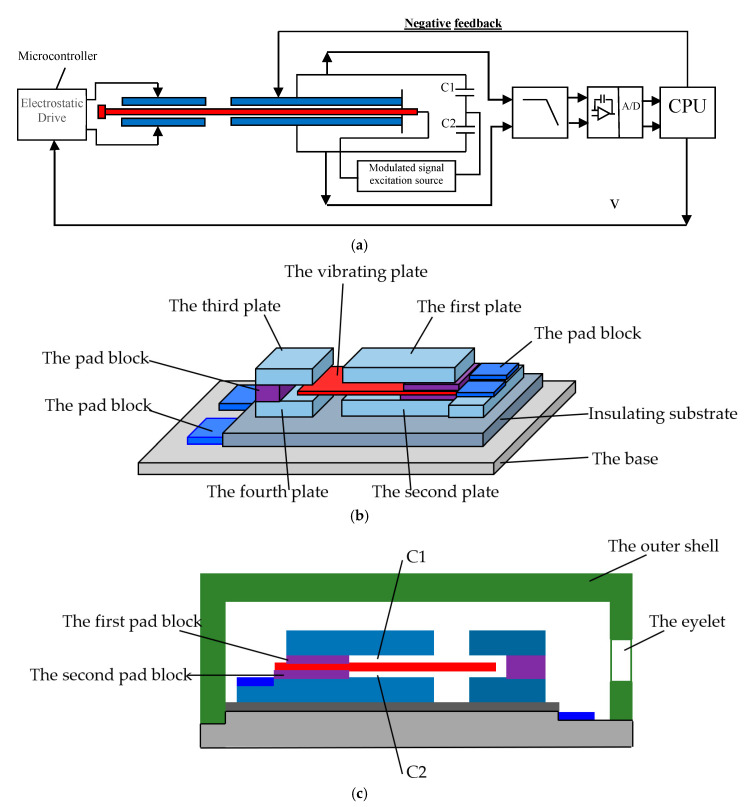
The sensor model with negative stiffness: (**a**) schematic diagram of sensor structure design; (**b**) the 3D structures of capacitive sensor with negative stiffness; (**c**) the 2D structures of capacitive sensor with negative stiffness.

It is difficult for general accelerometers to measure low-frequency signals, especially the signals below 1 Hz. Because the amplitude of acceleration at low frequency is relatively small, which means a weak excitation, and the low Signal Noise Ratio (SNR) makes the signal indistinguishable. In order to solve the above problem, the negative stiffness mechanism is adopted in this paper. The negative stiffness mechanism is that the greater the deformation of the system, the smaller the resistance. The proposed sensor model includes a variable capacitor and an electrostatic plate coupled with a micro controller as shown in Figure 2a–c.

Based on the principle of variable capacitance detection, the negative stiffness mechanism was employed by the proposed sensor. The negative stiffness sensor consists of two parts. The first part is a differential capacitor. The C1/C2 and capacitive sensors form a capacitance bridge which is driven by modulated AC signals. When the capacitance changes with acceleration, the capacitive reactance of the capacitance bridge will change. Then, the signal is amplified, filtered, and converted from analog to digital. A microprogrammed Control Unit (MCU) was adopted to make the system have better linear characteristics. The gap between capacitor chips should be small enough. Here, the negative feedback system was employed. When the acceleration is small in amplitude and frequency, the sensor detects very small outputs. To amplify these faint outputs, the MCU outputs an electrostatic force to the end of the chip as:

(3) $$F_{e}=\frac{1}{2}\frac{C}{d_{z}}V^{2}$$

where $F_{e}$ is electrostatic force by MCU, *C* is capacitance, $d_{z}$ is the capacitance spacing, which is the distance between the two electrostatic plates that attract each other, *V* is voltage. $F_{e}$ provides attractiveness and naturally reduces $d_{z}$. When $d_{z}$ is smaller, $F_{e}$ will be larger, and the deformation of the cantilever beam vibrating plate will be larger. When vibrating plate is deformed, the Wheatstone capacitor bridge used as acceleration measurement will be more unbalanced, and the acceleration output will become larger. So, this electrostatic force enlarges the offset of the vibrating plate and amplifies the acceleration sensitivity.

The specific structural design is as follows:

The variable capacitors are placed on the insulating substrate in parallel with the electrostatic plate. The variable capacitors are comprised of the first plate, the cantilever vibrating plate and the second plate, respectively. The vibrating plate is cantilevered between the first plate and the second plate. The first capacitor C1 is formed between the first plate and the vibrating plate, and the second capacitor C2 is formed between the vibrating plate and the second plate. A pair of variable capacitance structures is formed by C1 and C2. These two capacitances are equal at origin, and can be expressed as

(4) $$C_{1}{=C}_{2}=\frac{\varepsilon_{r}\varepsilon_{0}S}{d}$$

where *d* is the distance between the two plates, $\varepsilon_{r}$ and $\varepsilon_{0}$ are the relative permittivity of the medium and vacuum, respectively, $\varepsilon_{0}=8.85\times 10^{-12}\;F/m$, *S* is the overlapping area.

**Figure 3:**
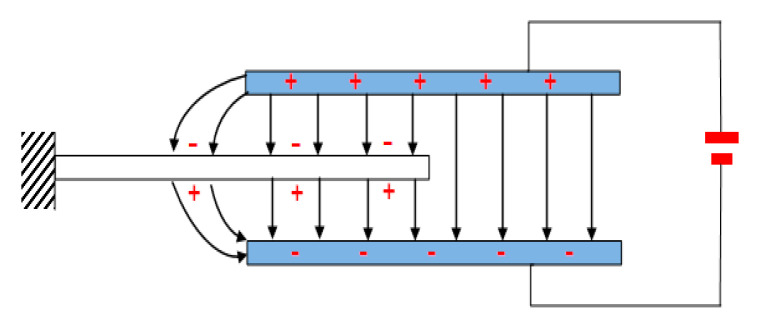
The electrostatic field.

The electrostatic plates are the third plate and the fourth plates, which are arranged on the insulating substrate and arranged parallel to each other. The free end of the vibrating plate is inserted between the third plate and the fourth plate, and the micro controller is coupled to the electrostatic plates to adjust the strength of electrostatic field between the third plate and the fourth plates. The two surfaces of the vibrating plates are polarized by the electric field shown in Figure 3.

**Figure 4:**
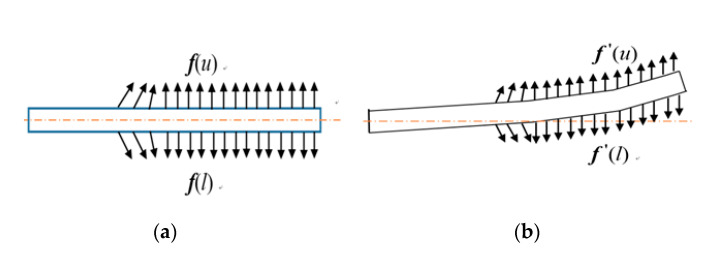
The electric filed forces on the vibrating plate: (**a**) The electric forces on the plate at rest (**b**) The electric forces on the deflected plate.

When the deflection of the vibrating plate does not occur as shown in Figure 4a, the two capacitances are the same, so there is no output. The capacitance values of the pair of capacitors C_1_ and C_2_ do not change and the output signals from the pair of capacitors is zero.

According to Coulomb’s law, the electric force (Coulomb force) between two charges *q*_i_ and *q*_j_ is expressed as:

(5) $$F=\frac{1}{4\pi \varepsilon_{0}}\frac{q_{i}\cdot q_{j}}{r^{2}}$$

where *r* is the distance between the two charges.

From Equation (5), it is noted that the electric force (Coulomb force) is inversely proportional to the square of the distance between the two charges. Therefore, the electric field forces at the upper and lower surface of the vibrating plate, denoted by ***f*** (*u*) and ***f*** (*l*), respectively, are equal. However, due to the deflection of the vibrating plate at vibrating time, the vibrating plate moves to near the upper plate as shown in Figure 4b. Then, the electric field forces at the upper surface of the vibrating plate are larger than those at the lower surface due to the smaller relative distance of the vibrating plate, i.e., $f^{\prime }\left(u\right)>f^{\prime }\left(l\right)$ in Figure 4b. So, the displacement of the vibrating plate is further amplified, and the position change of the vibrating plate with respect to the first and second plates will be enlarged, the stiffness of the vibrating plate is reduced. Therefore, the differences of capacitance values of C_1_ and C_2_ are enlarged, which causes the output signals of the variable capacitor to be amplified.

The strength of the electrostatic field can be adjusted through the micro controller coupled to the electrostatic plate in a feedback control fashion. The electrostatic force (Equation (3)) output by the MCU will reduce the distance between the two electrostatic plates, which will further enhance the strength of the electrostatic field. The stronger the electrostatic field the larger the amplification in the deflection of the vibrating plate, thus, the stronger the output signals. Therefore, when the output signals of a pair of variable capacitors are weak, the feedback mechanism will be activated, and the electrostatic force applied by the microcontroller on the electrostatic plate will increase, thereby enhancing the weak signals. Then, if the stiffness is further reduced, the resultant force of the electric field forces will be further enlarged, and the deflection of the vibrating plate will be further amplified. On the contrary, when the output signals from the pair of variable capacitors are not weak, the increase of the current applied to the electrostatic plate is reduced by the micro controller through the feedback mechanism, leading to the reduction in both negative stiffness effect and the amplification of output signals.

What needs illustration is that feedback control is to report back the displacement value of the electrostatic plate to the microcontroller, namely the capacitance value. In this sensor design, the standard for distinguishing high frequency input from low frequency input is the minimum resolution with no negative stiffness. When the system cannot resolve the acceleration value, the system will start the negative stiffness module to make the deviation larger.

The amplitude-frequency characteristics of the proposed sensor model with negative stiffness can be expressed by:

(6) $$\mu =\frac{A_{m}}{A_{s}}=\frac{1}{\sqrt{{\left[1-{\left(\frac{\omega }{{\omega^{\prime }}_{n}\left(A\right)}\right)}^{2}\right]}^{2}+{\left[2\zeta \left(\frac{\omega }{{\omega^{\prime }}_{n}\left(A\right)}\right)\right]}^{2}}}$$

where A is level of input excitation, ω is the excitation frequency, ${\omega^{\prime }}_{n}\left(A\right)$ is the variable natural frequency of the system. In the low frequency range, when the system cannot resolve the acceleration value, the system will start the negative stiffness module to make the deviation larger. So, when the excitation frequency changes in low frequency range, the resonance frequency of the system is not a constant value. The purpose of the negative stiffness is to add a large non-linear mass through electrostatic drive control, thereby reducing the overall resonance frequency of the cantilever beam and performing non-linear amplification.

In summary, based on the feedback control of the electric field, both the negative stiffness and amplification effects of the sensor model are adaptive, leading to the high sensitivity to weak and low frequency input and low sensitivity to high input.

## 3. The Design and Manufacture of the Proposed Sensor Prototype

### 3.1. The Design of the Proposed Sensor Prototype

**Figure 5:**
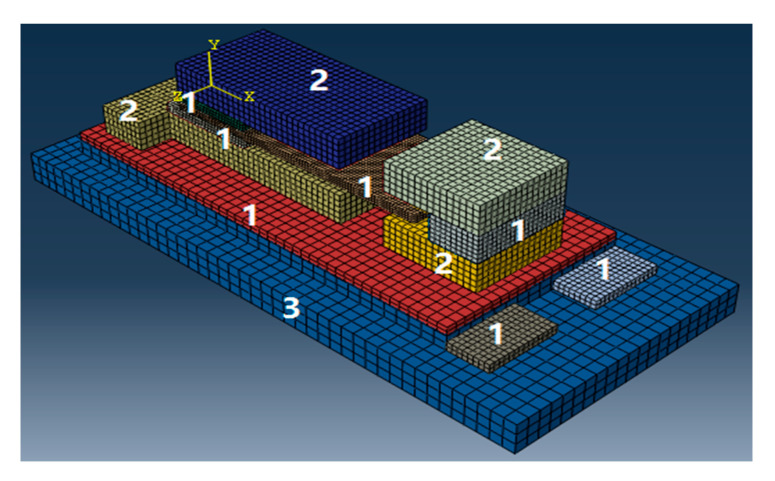
The selection of the materials.

As shown in Figure 5, the first to the fourth plates are made of Beryllium bronze. The vibrating plates are made of resin film (Corning E-XG glass), whose upper and lower surface are laminated with a conductive substance by sputtering. The first and second pads are insulators and are made of silica or other insulating materials with better stiffness. The material parameters are listed in Table 1.

### 3.2. The Manufacture of the Proposed Sensor Prototype with Packaging

**Figure 6:**
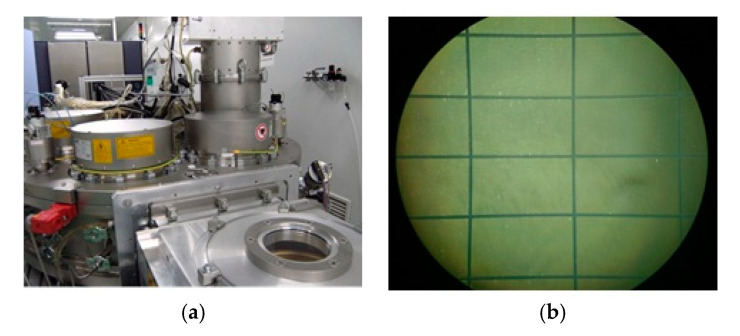
The key manufacturing process (**a**), Inductively coupled plasma (ICP) deep silicon etching; (**b**) Laser cutting.

The manuscript of proposed sensor prototype with negative stiffness is implemented by Micro-Electro-Mechanical Systems (MEMS) technology in the FATRI (Xiamen) Technologies Co., Ltd. The main manufacture procedures can be divided into: wafer cleaning → wafer lacquering technics → photolithography → plating → photoresist stripping → bonding techniques → scribing → chip packaging. The two key manufacturing processes are shown in Figure 6. Figure 6a is inductively coupled plasma (ICP) deep silicon etching technology which is a kind of high degree plasma etching, and is an effective machining method for silicon grooves with high aspect ratios. The Figure 6b is laser cutting technology.

**Figure 7:**
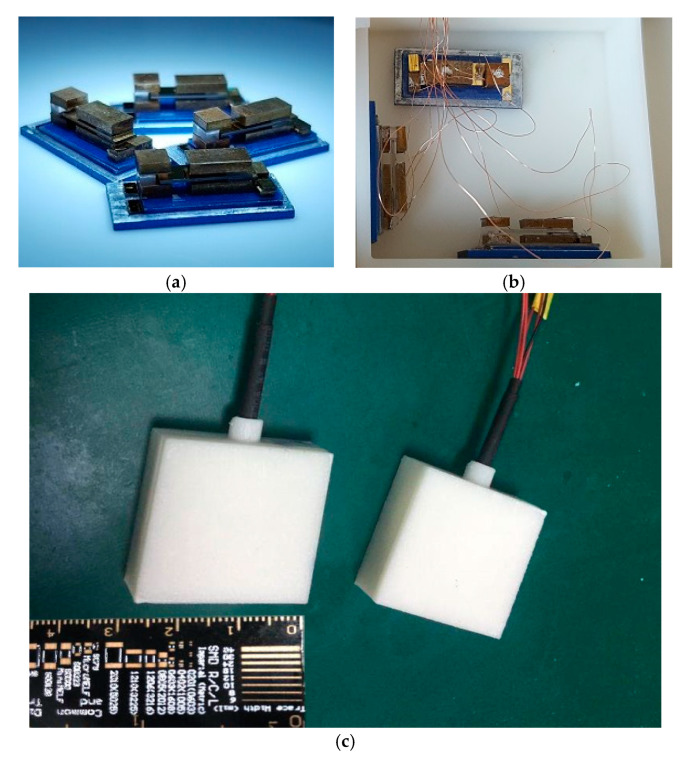
The packaging of sensor prototype. (**a**) The sensor units (**b**) The sensor units are placed in three directions (**c**) The packaging and the size of sensor prototype.

Then, the bare chip is assembled and packaged for the entire triaxial acceleration sensor. The size of the developed sensor after encapsulation is approximately 30 × 30 × 10 mm and weighs about 20 g. The sensor prototype comes with a ruler for reference shown in Figure 7.

The output of the proposed capacitance sensor is voltage (V). The relationship between output voltage and acceleration needs to be calibrated. Since the sensor is fully sealed, it is not affected by moisture. In addition, the proposed sensor is temperature corrected by Negative Temperature CoeffiCient (NTC).

Therefore, the proposed sensor prototype is small in size, lightweight and consumes less power. The sensor prototype is also well packaged, which means it can be used for lab and in-field testing.

### 3.3. Post-Signal Processing

The signal acquisition device directly collected signals accompanied by a variety of noise. The original signals should be noise-reduced to amplify the low-frequency and weak signals. The purpose of filtering was to reduce some high frequency noise and ripples. In general, the filter point was 10 times the sensor’s maximum operating frequency. In this proposed sensor, the maximum operating frequency was set as the frequency corresponding to half of the sensitivity attenuation according to the measurements of demand and effect. From the results in Section 4, the maximum operating frequency of the proposed sensor was 160 Hz, and the filter point was 1600 Hz.

## 4. Preliminary Testing

Some Preliminary Testing of the developed prototype of sensor was conducted in the lab at the FATRI Technologies Co., Ltd., Xiamen, China.

**Figure 8:**
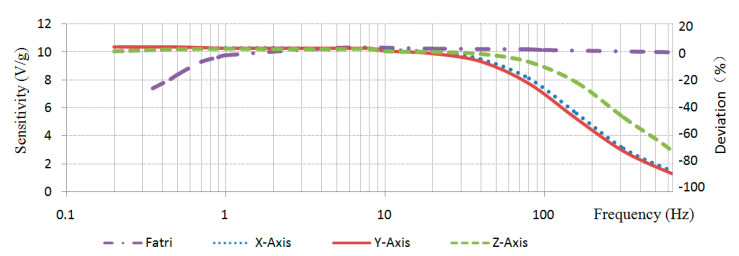
The variations of sensitivities with the frequency of inputs.

**Figure 9:**
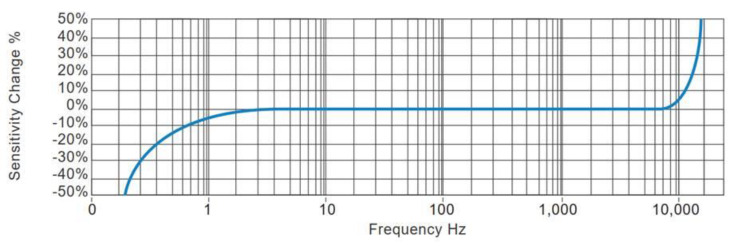
The typical frequency response of accelerometer by Hansford sensor Co., Ltd.

The adaptive sensitivity of the sensor prototype to the frequencies of inputs with 0.1 g amplitudes is shown in Table 2 and Figure 8. The dot dash line in the Figure 8 represents the positive stiffness sensor produced by FATRI as shown in Figure 1, while the other three lines represent the three directions of the proposed negative stiffness sensor. In the test range of 0–640 Hz, the negative stiffness sensor maintained a good sensitivity (10 V/g) to the low-frequency range (0.2–10 Hz) signals, because the faint signals of low frequency were adaptively amplified, which solved the problem of low sensitivity of the positive stiffness sensor in the low-frequency range. At the same time, the sensitivity to high frequency signals (>20 Hz) gradually decreased, indicating that the negative stiffness sensor was more sensitive to low frequency signals. Further comparison of sensors from other companies was added. Figure 9 is the typical frequency response of the accelerometer by Hansford sensor Co., Ltd., which had the same characteristics as the acceleration sensor by FATRI.

**Figure 10:**
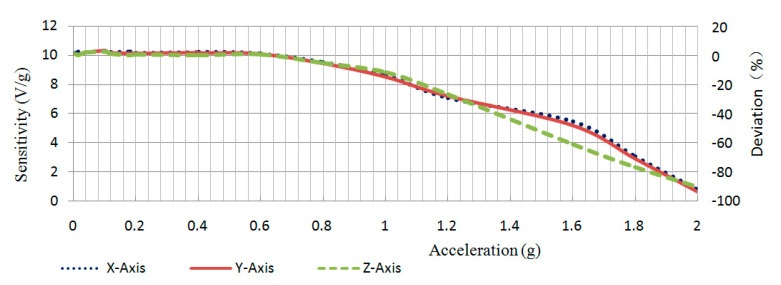
The plot of adaptive sensitivities with the amplitude of inputs.

For the variations of sensitivity of the sensor prototype with the amplitudes of the acceleration input, Table 3 and Figure 10 show the results when the input frequency was 1.0 Hz. In the test range of 0.004–2 g, the negative stiffness sensor maintained a good sensitivity (10 V/g) to the weak signals (0.004–0.6 g) because the weak signals were adaptively amplified. Meanwhile, the sensitivity to strong signals (>0.6 g) gradually decreased, which indicates that the negative stiffness sensor was more sensitive to weak signals.

Therefore, the developed sensor prototype has a calibration sensitivity of 10 V/g and a wide measurement frequency range. From the above testing results, it is shown that the sensitivities are higher to weak vibration than those to the strong vibration, which demonstrates that the sensor has adaptive amplification characteristics with high sensitivity to weak and low frequency input and low sensitivity to high inputs.

## 5. Preliminary Experimental Monitoring Sand Interfacial Slide by the Sensor Prototype

Due to sand dunes and loess or loose soil having similar characteristics: loose texture, large pores, good water permeability, liable to collapse, loess was replaced by sand in the landslide testing. Furthermore, cement board and mudstone/rock having the same characteristics of poor water permeability, cement board was chosen as the bottom rock in the testing.

**Figure 11:**
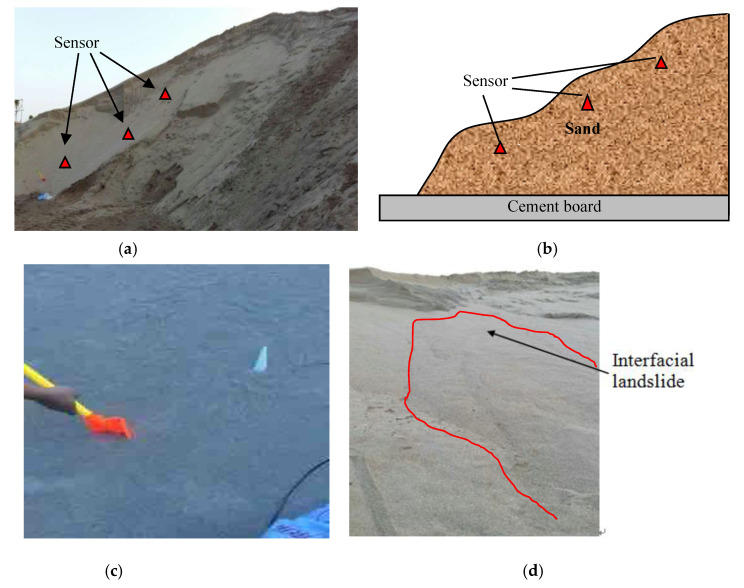
Experimental monitoring of sand interface slide by the sensor prototype. (**a**) The sand dune in field; (**b**) Schematic diagram of sand pile and sensor location; (c) Disturbance; (d)The sand interface slide due to disturbance.

The developed sensor prototype with negative stiffness was deployed on the surface of the sand dune as shown in the Figure 11. The sand dune was slightly disturbed to simulate the sand interfacial slide. In the landslide experiments, the sensors were embedded in the lateral range of the possible landslide area. In addition, a small disturbance was made to the surface of the sand dune, and the resulting landslide only occurred on the shallow surface of the sand. The sensors did not generate motion.

**Figure 12:**
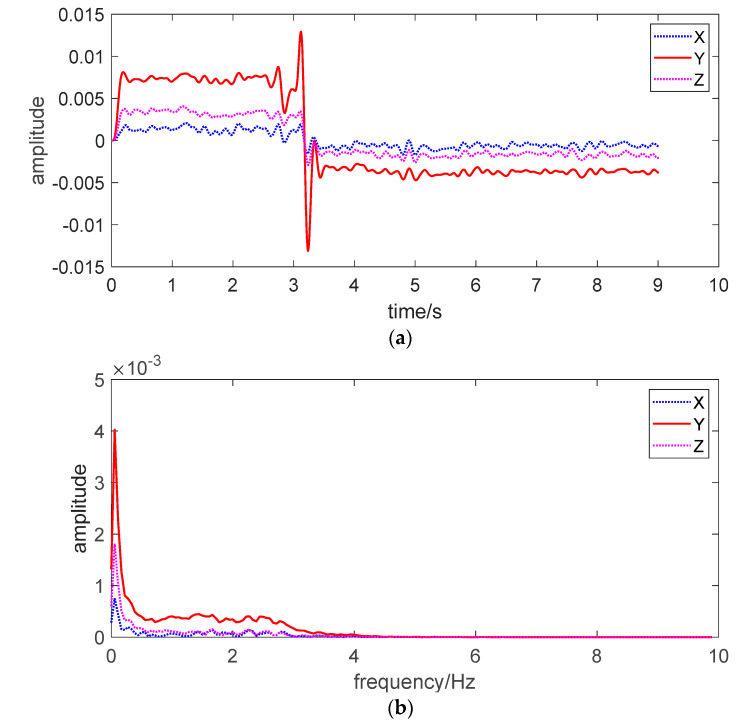
The monitoring results of sand interface slide in the experiment. (**a**) Time history of the recorded signals (**b**) Spectra of the recorded signals.

The Figure 12a is the time history of the recorded signals by the sensor prototype, the Figure 12b is the spectra of the recorded signals. The analysis of the signals in Figure 12a showed that as soon as the small disturbance was generated, the sand squirm in the initial stage of the landslide would occur. In the first set of experiments, the moment that the sand began to squirm was 0.0136 s. As the *Y*-axis was along the direction of the landslide, noise signals could be seen in the initial stage of the landslide even if there was no macro landslide. The initial squirm was slow and the range was small, in which the Y-direction acceleration was the largest. After that, the peristaltic velocity increased rapidly and the range became larger. Sand squirm became sliding, and a large area of sand produced landslide. The rise of the monitoring curve was the maximum acceleration of the landslide. Because the sand grains were not cohesive and were dry sand, the sand landslide was completed in an instant, and the abrupt descent of the monitoring curve was the deceleration after the landslide was completed, and then the sand grains squirmed slowly. It is shown in the Figure 12b that the frequency corresponding to the maximum vibration acceleration generated by the sand body landslide was about 0.1 Hz. From the output signals from the sensor prototype package in Figure 12, it is clearly shown that the occurrence of sand interface slide can be monitored by the significant changes in the time history of the recorded signals by the sensor prototype.

## 6. Conclusions

In this paper, a novel sensor prototype with enhanced and adaptive sensitivity to weak and low frequency vibrations was developed for monitoring of loess–mudstone/soil-rock interfacial landslides. The novelty of the sensitive sensor is based on variable capacitances and negative stiffness implemented by an embedded micro controller for adjustable electrostatic field and electric filed forces through feedback control. Such a sensor has been designed and manufactured by MEMS including proper packaging of a prototype. Some preliminary tests illustrate the enhanced and adaptive sensitivity of the senor prototype with high sensitivity to weak and low frequency input and low sensitivity to high input. Moreover, the preliminary experimental results of monitoring sand interfacial slide demonstrate the promising application of the developed sensor prototype for early warning of soil-rock interfacial landslide.

As these are preliminary studies of such a sensitive sensor prototype, more investigations are required to improve the performance of the sensors and conduct in-field testing of the developed sensor for monitoring soil-rock interfacial landslides.

## Figures and Tables

**Table 1:** The material parameters.

No.	Material	Density (kg/m^3^)	Elastic Modulus (GPa)	Poisson’s Ratio
1	Resin film	2230	71.7	0.21
2	Beryllium bronze	8260	110	0.35
3	AL6061	2200	65	0.17

**Table 2:** The variations of sensitivities with the frequency of the acceleration.

Frequency (Hz)	Sensitivity (V/g)		
	X	Y	Z
0.2	10.07	10.38	10.05
0.5	10.22	10.37	10.22
1	10.29	10.29	10.24
2	10.28	10.28	10.22
4	10.27	10.27	10.19
8	10.23	10.25	10.22
10	10.15	10.08	10.06
20	10.01	9.91	10.01
40	9.51	9.32	9.87
80	8.11	7.75	9.31
160	5.62	5.2	7.83
315	3.08	2.88	5.33
630	1.44	1.28	2.87

**Table 3:** The adaptive sensitivities with the amplitudes of inputs.

Acceleration (g)	Sensitivity (V/g)		
	X	Y	Z
0.004	10.15	10.08	10.10
0.015	10.22	10.06	10.02
0.04	10.19	10.19	10.18
0.1	10.29	10.29	10.24
0.12	10.21	10.17	10.11
0.18	10.23	10.13	10.02
0.2	10.15	10.12	10.06
0.4	10.21	10.14	10.01
0.6	10.11	10.08	10.07
0.8	9.51	9.42	9.46
1.0	8.62	8.52	8.83
1.2	7.08	7.18	7.33
1.6	5.44	5.18	3.87
1.8	3.03	2.9	2.31
2.0	0.74	0.63	0.96
